# Exploring the Relationship between Self-Compassion and Psychological Pain: A Canonical Correlation Analysis

**DOI:** 10.3390/bs14080631

**Published:** 2024-07-24

**Authors:** Ariana Garabedian, Alexandra Dluzniewski, Russell T. Baker, Madeline P. Casanova

**Affiliations:** 1 WWAMI Medical Education Program, University of Idaho, Moscow, ID 83844, USA; agarab@uw.edu; 2 Idaho Office of Rural and Underserved Medical Research, University of Idaho, Moscow, ID 83844, USA; adluz@uidaho.edu (A.D.); russellb@uidaho.edu (R.T.B.)

**Keywords:** positive psychology, self-compassion, psychological pain, canonical correlation

## Abstract

This study investigated the association between self-compassion and psychological pain across various demographic variables. Using canonical correlation analysis, we observed an inverse relationship between the combined factors of the Self-Compassion Scale (SCS) and the Orbach and Mikulincer Mental Pain Scale (OMMP-8). Subgroup analyses revealed differences in SCS subscales among demographic groups with females, individuals with mental health diagnoses, and non-athletes displaying higher scores on negative SCS subscales and PsyPn. Injury status did not significantly affect self-compassion levels, although injured individuals scored higher on the irreversibility subscale of PsyPn. Negative SCS factors exhibited larger group differences and stronger correlations with PsyPn, indicating the potency of negative thinking in influencing psychological pain. These findings underscore the importance of self-compassion in mental health and suggest potential implications for intervention strategies.

## 1. Introduction

Mental health is a pressing concern in the United States, where approximately 50 million adults experienced a mental illness in 2019 [1]. Tragically, suicide continues to be a significant contributor to mortality in the United States, particularly among those aged 10–14 years and 25–34 years, ranking as the second leading cause of death [2]. Additionally, more than one million suicide attempts were reported in 2022 [3]. The intricate link between suicide, depression, and psychological pain (PsyPn) has been extensively researched, with PsyPn defined as an overwhelming negative emotional state encompassing feelings of self-inadequacy and isolation from others [4]. Researchers have also characterized it as a sense of mourning and hopelessness following emotional trauma or loss of essential needs [5]. Given the profound association between PsyPn and mental health, there is a critical need to explore factors that may mitigate or reduce PsyPn.

One such factor with an inverse relationship with psychopathology (i.e., a generalized term that encompasses mental health symptoms) is self-compassion [6]. Self-compassion, as conceptualized by [7], involves being open to one’s own suffering, not avoiding it, and fostering self-healing through kindness. It encompasses a nonjudgmental understanding of one’s pain, inadequacies, and failures, recognizing that suffering is part of the human experience [7]. Researchers have found that individuals who report greater self-compassion exhibit lower levels of depression and anxiety symptoms [6]. Self-compassion is considered a protective factor against negative mental health outcomes and is positively correlated with well-being, happiness, and coping [8].

To measure self-compassion, Neff developed the Self-Compassion Scale (SCS), which assesses three facets of self-compassion: self-kindness, common humanity, and mindfulness [7]. Researchers have assessed the psychometric properties of the SCS and found good internal consistency (α = 0.75 to 0.81) and test–retest reliability (α = 0.80 to 0.88; [7]). Additionally, an excellent fit across 20 samples was found for the six-factor model using confirmatory factor analysis (CFA) and exploratory structural equation modeling (ESEM) analysis techniques [9]. Additionally, its inverse relationship with depression, anxiety, stress, and maladaptive psychological states (i.e., perfectionism, rumination, pain, catastrophizing, and self-criticism) [9,10,11,12], coupled with a positive relationship with well-being, self-esteem, life-satisfaction, emotional intelligence, and social connectedness underscores its multifaceted importance [10,11,12].

While the inverse relationship between self-compassion and depression has been studied [12], the relationship between self-compassion and PsyPn remains unexplored. Recognizing that self-compassion may serve as a tool for emotional regulation [13], potentially mitigating emotional distress associated with PsyPn, this study aimed to elucidate the relationship between self-compassion and PsyPn by using canonical correlation analysis (CCA). Further, it sought to examine how this relationship manifested among different demographic groups, offering valuable insights into potential nuances in the relationship between self-compassion and mental well-being.

## 2. Materials and Methods

Upon receiving Institutional Review Board (IRB) approval, participants were recruited using a combination of snowball and convenience sampling methods [14]. An electronic survey was developed using Qualtrics software, Version March 2020 (Qualtrics Inc., Provo, UT, USA) that included the SCS, the OOMP-8, and a participant demographic questionnaire. A link was sent to personal contacts of the research team and advertised on social media pages. A volunteer platform, ResearchMatch [15], was also used to recruit participants. Finally, participants provided informed consent prior to completing the survey.

### 2.1. Instrumentation

#### 2.1.1. Self-Compassion Scale

To assess self-compassion, the SCS was used [7]. The SCS is a 26-item instrument designed to assess six factors: self-kindness (5 items), self-judgement (5 items), common humanity (4 items), isolation (4 items), mindfulness (4 items), and over-identification (4 items). The SCS has well established psychometric properties: good internal reliability (α = 0.75 to 0.80), test–retest reliability (α = 0.80 to 0.88), predictive validity, and good model fit across 20 samples [7,10,16,17,18]. Participants used a 5-point Likert scale (1 = almost never; 5 = almost always) to respond to items. Six factor scores were created by summing all items in each factor and these factor scores could range from 5 to 25 or 4 to 20 depending on the number of items in each factor [19]. In the current study, scores were not reversed for the self-judgement, isolation, or over-identification factors, with high scores indicating high self-judgement (i.e., lower levels of self-compassion). Further, high scores on the more positive factors (e.g., self-kindness) also indicated a higher level of self-compassion.

#### 2.1.2. Psychological Pain Scale

To assess PsyPn, a short form version of the Orbach and Mikulincer Mental Pain Scale (OMMP) was used [20]. The OMMP-8 [20] is an 8-item instrument that assesses three factors of PsyPn: experience of irreversibility (IRR; two items), emotional flooding (EF; three items), and narcissistic wounds (NW; three items). The three-factor model was established using CFA techniques and excellent fit was demonstrated along with sound construct validity [20]. Participants used a 5-point Likert scale (1 = strongly disagree, 2 = disagree, 3 = agree to some extent, 4 = agree, 5 = strongly agree) to answer each item. Factor scores were calculated by summing all items in each factor and scores could range from 2 to 10 or 3 to 15 depending on the number of items in each factor.

#### 2.1.3. Participant Demographic Questionnaire

A participant questionnaire was created to collect demographic variables of interest. Variables included sex, ethnicity, age, highest level of education, physical activity level, prior or current diagnosis of a mental illness, and musculoskeletal injury status.

### 2.2. Data Analysis

Data were exported from the Qualtrics survey to the Statistical Package for Social Sciences Version 27 (SPSS, Inc., Chicago, IL, USA) for analysis. Participants missing more than 10% of the SCS or OMMP-8 were removed from the dataset; and any remaining missing data was replaced with the rounded mean score [21]. After missing data were calculated, data normality was assessed using histograms and skewness and kurtosis values. Univariate outliers were assessed using z-scores (cutoff value of |3.3|) and multivariate outliers were assessed using Mahalanobis distance (*p*-value = 0.01) [21,22].

#### 2.2.1. Mean Score Analysis

Mean scores were calculated for the subscales of the SCS and the OMMP-8 for the full sample and by subgroups of interest, which were categorized by sex (i.e., males, females), athlete status (i.e., individuals who are physically active, individuals who are not physically active), mental health diagnosis (i.e., individuals who have a past or current mental illness diagnosis, individuals who do not have a history of a mental illness diagnosis), and injury status (i.e., individuals who have an acute, subacute, persistent, or chronic injury and individuals who do not have a musculoskeletal injury). Mean scores were compared using mean differences (i.e., unstandardized effect size) and Cohen’s d standardized effect sizes; Cohen’s d scores were assessed using the guidelines of d = 0.2 as a small effect size, d = 0.5 as a medium effect size, and d = 0.8 as a large effect size [23].

#### 2.2.2. Canonical Correlation Analysis

Canonical correlation analysis (CCA) was conducted to assess the pattern of relationships between self-compassion subfactors and PsyPn subfactors. Assumptions of linearity, multivariate normality, and homoscedasticity were assessed by examining a bivariate scatterplot of the canonical variate scores [22,24]. To determine canonical correlation set interpretation, the following were assessed [22,24,25]: the *p*-value of Wilk’s λ, the inverse of Wilk’s λ, eigenvalue, canonical correlation, and the percentage of variance explained. Wilk’s λ (*p* < 0.01) would be considered statistically significant; however, *p*-value is sensitive to sample size. Therefore, we also assessed the inverse of Wilk’s λ to calculate the amount of variance unaccounted for; this is commonly interpreted as a measure of effect size [24]. Therefore, the inverse of Wilk’s λ was assessed using the guidelines of d = 0.2 as a small effect size, d = 0.5 as a medium effect size, and d = 0.8 as a large effect size [23]. Canonical correlation sets with R > 0.30, accounting for more than 10% of the variance, and eigenvalues > 1 were considered meaningful. Further, canonical loadings greater than 0.71 (50% overlapping variance) were considered excellent, 0.63 (40% overlapping variance) very good, 0.55 (30% overlapping variance) good, 0.45 (20% overlapping variance) fair, and 0.32 (10% overlapping variance) poor [25].

## 3. Results

A total of 1360 individuals completed the SCS and the OMMP-8, and 179 outliers were identified and removed from the dataset, resulting in 1181 participants remaining for analysis. Participants had a mean age of 40 years (SD = 16, range = 18 to 88 years) with 80.0% (*n* = 920) reporting their sex as female and 19.4% (*n* = 223) as male. Three-fourths of the sample (74.9%) reported having a bachelor’s degree or higher, with almost half of the sample holding a graduate degree (48.4%). Nearly half of the sample (44.3%) indicated they had a prior or current mental illness diagnosis, while most (53.4%) indicated that they did not have a past or current mental illness diagnosis. Most of the sample was Caucasian/White (83.3%), while African American/Black, Hispanic or Latino, and Asian/Pacific Islander each represented about 5% of the remaining sample. Most of the sample reported low to medium levels of physical activity (70.7%), with recreational athletes representing 36.9% of the sample. Furthermore, more than half (61.1%) of the sample reported not having a musculoskeletal injury, while 34.4% had an injury classified as chronic or persistent. A full breakdown of demographics is presented in Table 1.

### 3.1. Mean Score Analysis

Mean scores and standard deviations were calculated for each subscale of the SCS and OMMP-8 (Table 2). Chronbach’s alpha was calculated for both the OMMP-8 (α = 0.87) and SCS (α = 0.75). Additionally, mean scores for each subscale of the SCS and OMMP-8 by subgroup are reported in Table 3 along with mean differences and Cohen’s d values reported in Table 4. Females scored higher on self-judgement, isolation, and over-identification compared to males on the SCS, with smaller differences on the positive factors; while females scored higher across the three factors of the OMMP-8, the largest sex difference was found in the emotional flooding factor (Table 3 and Table 4). Overall, athletes reported higher levels of self-compassion and lower OMMP scores compared to non-athletes (Table 3 and Table 4). Individuals with a mental health diagnosis reported higher mean scores on self-judgement, isolation, and over-identification, and lower scores on self-kindness, common humanity, and mindfulness on the SCS, as well as higher scores on the OMMP-8 compared to individuals without a mental health diagnosis (Table 3 and Table 4). Lastly, the injury subgroup demonstrated small to negligible mean differences for all subscales of the SCS and the OMMP-8, except for the irreversibility subscale, with individuals with a musculoskeletal injury reporting higher mean scores on the OMMP-8 (Table 3 and Table 4).

### 3.2. Canonical Correlation Analysis

A CCA was performed to assess the pattern of relationships between self-compassion and PsyPn subfactors. Assumptions of homoscedasticity, linearity, and multivariate normality were met. A strong positive canonical correlation (R = 0.73, *p* ≤ 0.001) between the SCS subscales and the OMMP-8 subscales, which accounted for 53% of the variance, was found. For CC Set 1, Wilk’s *λ* was 0.43 (F(18) = 63.54, *p* < 0.001; Table 5) with the inverse of Wilk’s *λ* = 0.57, indicative of a medium effect size. For CC Set 2, Wilk’s *λ* was 0.919 (F(10) = 10.11, *p* < 0.001; Table 5) with the inverse of Wilk’s *λ* = 0.08, indicative of a very small effect size. For CC Set 3, Wilk’s *λ* was 0.978 (F(4) = 6.73, *p* < 0.001; Table 5) with the inverse of Wilk’s *λ* = 0.02, of a negligible effect size. Of the three canonical correlations, only CC Set 1 had an eigenvalue greater than one. CC Set 1 also had a correlation greater than 0.3 and accounted for more than 10%; therefore, CC Set 1 was interpreted (Table 6). While CC Set 2 did not meet most a priori criteria, its loadings were greater than 0.4, which was considered good and may offer meaningful relationships. Lastly, CC Set 3 was statistically significant, but its effect size was small and it did not meet any other recommended criteria.

Examination of the loadings indicated there was a relationship between all SCS subscales and the OMMP-8 subscales. Specifically, CC Set 1 loadings suggested a strong relationship between self-judgement, isolation, over-identification, and emotional flooding and CC Set 2 indicated that common humanity was inversely related to narcissistic wounds.

## 4. Discussion

The purpose of our study was to explore the relationship between self-compassion and PsyPn by using CCA and how this relationship manifested across various demographic variables (i.e., sex, diagnosis of a mental illness, and injury status) by examining mean differences. We found a significant relationship between the multivariate combination of the SCS and OMMP-8 factors. Importantly, our findings underscored a strong inverse relationship between self-compassion and PsyPn. Individuals who scored higher on the SCS exhibited lower levels of PsyPn, while those who reported lower SCS scores exhibited higher levels of PsyPn. Additionally, subgroup analyses revealed mean differences across the six SCS subscales between subgroups.

Our findings are congruent with previous research that identified the inverse relationship between self-compassion and adverse mental health outcomes, such as depression and anxiety [6,12]. Moreover, our findings resonate with prior research demonstrating a positive association between self-compassion and well-being, emotional intelligence, and self-esteem [8]. Notably, our study extends this understanding by revealing an inverse relationship of positive SCS factors (self-kindness, common humanity, mindfulness) with PsyPn, as measured with the OMMP-8. Further, CC Set 1 indicated that the negative SCS factors (self-judgement, isolation, over-identification) were most strongly related to the emotional flooding subscale of the OMMP-8. This could suggest that addressing emotional flooding may be beneficial. While core beliefs like narcissistic wounds and perceptions of irreversibility can be addressed through cognitive behavioral therapy (CBT) techniques such as self-talk [26], emotional flooding often points to nervous system dysregulation (e.g., fight-or-flight activation) [27]. Therefore, implementing body-based techniques like mindfulness and deep breathing may be beneficial for individuals with low self-compassion. Conversely, CC Set 2 indicated that common humanity and narcissistic wounds exhibited a strong inverse relationship. CC Set 2 loadings should be interpreted with caution as it did not meet all a priori criteria; however, its loadings suggested that there was overlapping variance among the variables [24].

We also observed variations in the strength of the relationship between self-compassion and PsyPn across demographic groups (i.e., sex, athlete status, mental health diagnosis, and injury status). Females displayed higher scores on self-judgment, isolation, over-identification, and emotional flooding, reflecting consistent trends of higher pain intensity and chronic pain incidence reported by women [28]. Though physical pain is distinct from psychological pain, these variables are thought to be related through neural networking [29], offering explanation for our findings. However, there is also evidence that females tend to demonstrate higher pain acceptance, while males were reported to have higher levels of kinesiophobia, more mood disturbances, and lower activity levels than females [30]. Therefore, the experience of pain is nuanced, and future research is warranted to understand sex differences in pain acceptance and relevant psychological factors.

In the current study, individuals with a mental health diagnosis scored lower on the SCS (i.e., higher on the negative subscales and lower on the positive subscales) and higher on the OMMP-8 (i.e., high PsyPn) compared to individuals without a mental health diagnosis, indicating potential challenges in enacting self-compassion among these individuals. It is possible that individuals with a diagnosis of depression or anxiety, while able to objectively define compassion, have difficulty enacting self-compassion because of the self-described negative impacts of their psychological disorder [31]. Our findings align with prior research, emphasizing the importance of self-compassion in promoting mental health; however, further research is needed to establish best practice recommendations and interventions for addressing self-compassion in individuals with mental illness.

Another interesting finding is that athletes reported lower scores on the negative (i.e., self-judgment, isolation, and over-identification) SCS subscales and lower scores on the OMMP-8 relative to non-athletes. This may be partially attributed to learned methods of decreasing burnout in competitive athletics [32] that increase self-compassion and mindfulness. This finding also adds to literature suggesting physical activity contributes to positive mental health [33] and thus, prescribing physical activity to individuals for enhanced well-being should be further explored. Interestingly, injury status did not yield significant differences in self-compassion; however, there was a large effect size associated with the irreversibility subscale of the OMMP-8, where injured individuals scored higher. The two items that capture irreversibility are “The pain will never go away” and “I will never be able to reduce my pain”. What an individual believes about their pain is a strong predictor of how long the pain will persist [34]. This suggests that practitioners and clinicians may want to assess and understand how an individual is appraising an injury. This may be especially important for individuals experiencing more chronic injuries and thus chronic pain, which could be contributing to their psychological pain. This further supports the idea that physical and PsyPn are processed through similar neural networking [29]. A paucity of data exists regarding whether experiences of PsyPn and self-compassion differ among those with and without a musculoskeletal injury. Our study offers the novel insight that injured individuals experience self-compassion similarly to healthy individuals. In the context of sports injury, however, recent findings suggest that self-compassion is crucial in effectively coping with physical injury, especially short-term sports injury [35]. Therefore, self-compassion may have an impact on injury recovery, but injury status may not necessarily impact levels of self-compassion.

Interestingly, we found that the negative SCS factors had larger effect sizes between subgroups and had higher correlations with OMMP-8 factors compared to the positive SCS factors; meaning the negative SCS factors demonstrated larger group differences and a stronger relationship with PsyPn. The statement, “I am my own worst critic” is more of a phenomenon than an idiom, in that negative events are more potent, salient, and efficacious than positive events [36]. Our findings extend this idea, with PsyPn being more strongly related to the negative SCS factors. Further, this also offers a potential explanation for the larger differences observed in the demographic subgroups, where more vulnerable subsamples (e.g., mental health diagnosis, non-athlete) were prone to negative thinking and therefore more likely to score higher on the negative subscales of the SCS and PsyPn.

### Limitations and Future Research

Despite our study’s extensive sample, the predominance of female participants (80.0%) warrants caution in generalizing sex-based conclusions. The lack of longitudinal data and the omission of certain variables (e.g., sexual orientation, gender identity, chronic illness, socioeconomic status, ethnicity and cultural background, etc.) limit the depth of our analyses. Future research should include questions associated with PsyPn with regard to self-identified sexual orientation [37], gender identity [38], and family or household needs [39]. For example, researchers have reported that the transgender population experiences disproportionate negative mental health outcomes including PsyPn and suicidal ideation [37], and older adults with both parents still living and requiring support were more likely to experience PsyPn [39].

Furthermore, collecting more detailed mental health diagnosis information, treatment plans, and time since diagnosis could enhance our understanding of self-compassion in individuals with specific mental health conditions. Our study serves as a foundational exploration, urging future investigations to delve into novel areas such as social media exposure and its potential impact on the relationship between self-compassion and PsyPn across diverse demographic groups. For example, researchers have begun assessing the relationship between self-compassion and psychological well-being in patients with type 2 diabetes [40], undergraduate students during the COVID-19 pandemic [41], and healthcare providers [42]. Lastly, while our study provides valuable insights into the complex interplay between self-compassion and PsyPn, we did not assess the causal pathway between these two variables. Although PsyPn overlaps with several psychosocial concepts, many unmeasured variables and demographic nuances still warrant further exploration. This additional research is essential to refine our understanding of the potential causal pathway between self-compassion and PsyPn and to better inform targeted interventions.

## 5. Conclusions

Our findings revealed nuanced relationships between all SCS subscales and all OMMP-8 subscales across the demographic groups. However, a consistent inverse relationship between self-compassion and PsyPn was present across the sample: individuals with greater self-compassion tended to experience less PsyPn, underscoring the transformative potential of cultivating this trait. Thus, fostering self-compassion could be pivotal in mitigating emotional distress, especially for groups prone to heightened PsyPn. Future research should extend this inquiry to under-represented groups and explore behavioral interventions targeting PsyPn, contributing to a more nuanced understanding and practical applications of self-compassion in diverse populations.

## Figures and Tables

**Table 1:** Demographic characteristics of survey respondents.

Characteristic	*n* (%)
Sex	
Male	223 (18.9)
Female	920 (77.9)
Prefer not to answer	7 (0.6)
Unknown	31 (2.6)
Education	
Some high school, no diploma	2 (0.2)
High school or GED	41 (3.5)
Some college, no degree	150 (12.7)
Associate degree	68 (5.8)
Bachelor’s degree	304 (25.7)
Master’s degree	414 (35.1)
Doctoral degree	143 (12.1)
Other	27 (2.3)
Unknown	32 (2.7)
Mental Health Diagnosis	
Yes	509 (43.1)
No	614 (52.0)
Prefer not to answer	27 (2.3)
Unknown	31 (2.6)
Ethnicity	
Caucasian	984 (81.8)
African American	53 (4.4)
Hispanic	63 (5.2)
Asian/Pacific Islander	68 (5.7)
Other	35 (2.9)
Activity Level	
Inactive	216 (18.3)
Low	458 (38.8)
Medium	358 (30.3)
High	123 (10.4)
Unknown	26 (2.2)
Athlete Classification	
Competitive athlete	34 (2.9)
Recreational athlete	178 (15.1)
Occupational athlete	135 (11.4)
Activities of daily living	135 (11.4)
Unknown	2 (0.4)
Injury Status	
Healthy	705 (59.7)
Acute injury	23 (1.9)
Subacute injury	29 (2.5)
Persistent injury	114 (9.7)
Chronic injury	283 (24.0)
Unknown	27 (2.3)
Overall Health Status	
Excellent	134 (11.3)
Very good	417 (35.3)
Good	378 (32.0)
Fair	184 (15.6)
Poor	42 (3.6)
Unknown	26 (2.2)

**Table 2:** SCS and OMMP-8 scale descriptives. Means and standard deviations of the SCS and OMMP-8 subfactors.

Scale	M (SD)
SCS Subfactor	
Self-Kindness	14.32 (4.62)
Self-Judgement	15.79 (5.27)
Common Humanity	12.28 (3.82)
Isolation	12.04 (4.30)
Mindfulness	12.81 (3.58)
Over-Identification	12.01 (4.27)
OMMP-8 Subfactor	
Irreversible Pain	3.84 (1.94)
Emotional Flooding	8.26 (3.09)
Narcissistic Wounds	5.03 (2.04)

Note: SCS = Self-Compassion Scale; OMMP-8 = Orbach and Mikulincer Mental Pain Scale.

**Table 3:** SCS and OMMP-8 subgroup mean scores. Mean scores of the SCS and OMMP-8 subfactors by demographic variables of interest.

**SCS**	**Sex**		**Athlete Status**		**MH Diagnosis**		**Injury**	
Factor	Male M (SD)	Female M (SD)	Yes M (SD)	No M (SD)	Yes M (SD)	No M (SD)	Yes M (SD)	No M (SD)
Self-Kindness	14.22 (4.9)	14.36 (4.6)	14.98 (4.7)	13.88 (4.6)	13.39 (4.6)	15.15 (4.5)	13.99 (4.7)	14.57 (4.5)
Self- Judgement	13.79 (5.4)	16.20 (5.2)	14.73 (5.4)	16.48 (5.1)	17.57 (5.0)	14.15 (5.1)	16.30 (5.2)	15.40 (5.3)
Common Humanity	11.96 (3.8)	12.36 (3.8)	12.70 (3.9)	11.99 (3.8)	11.84 (3.8)	12.67 (3.8)	12.13 (3.8)	12.38 (3.8)
Isolation	10.48 (4.6)	12.38 (4.2)	11.04 (4.3)	12.74 (4.2)	13.50 (4.0)	10.72 (4.2)	12.57 (4.3)	11.69 (4.3)
Mindfulness	13.15 (4.0)	12.76 (3.5)	13.38 (3.7)	12.45 (3.5)	12.10 (3.5)	13.47 (4.1)	12.61 (3.6)	12.99 (3.6)
Over- Identification	10.06 (4.4)	12.41 (4.1)	11.11 (4.4)	12.61 (4.1)	13.46 (4.0)	10.68 (4.2)	12.33 (4.1)	11.76 (4.4)
**OMMP-8**	**Sex**		**Athlete Status**		**MH Diagnosis**		**Injury**	
Factor	Male M (SD)	Female M (SD)	Yes M (SD)	No M (SD)	Yes M (SD)	No M (SD)	Yes M (SD)	No M (SD)
Irreversible Pain	3.64 (1.9)	3.88 (2.0)	3.29 (1.6)	4.22 (2.1)	4.48 (2.1)	3.26 (1.6)	4.79 (2.2)	3.22 (1.5)
Emotional Flooding	7.39 (3.2)	8.45 (3.1)	7.60 (3.2)	8.71 (3.2)	9.30 (3.0)	7.30 (2.9)	7.94 (3.1)	8.73 (3.1)
Narcissistic Wounds	4.83 (2.0)	5.06 (2.0)	4.61 (1.9)	5.30 (2.1)	5.53 (2.2)	4.58 (1.8)	4.85 (2.0)	5.28 (2.1)

Note: SCS = Self-Compassion Scale; OMMP-8 = Orbach and Mikulincer Mental Pain Scale; M = mean; SD = standard deviation.

**Table 4:** SCS and OMMP-8 subgroup mean differences and Cohen’s d. SCS and OMMP-8 subfactor mean score differences and Cohen’s d by demographic variables of interest.

**SCS**	**Sex**		**Athlete Status**		**MH Diagnosis**		**Injury**	
Subfactor	MeanDiff	Cohen’s d	MeanDiff	Cohen’s d	MeanDiff	Cohen’s d	MeanDiff	Cohen’s d
Self-Kindness	0.13	0.03	1.10	0.24	1.76	0.39	0.6	0.13
Self-Judgement	2.40	0.46	1.75	0.34	3.41	0.68	0.9	0.17
Common Humanity	0.40	0.10	0.71	0.19	0.84	0.22	0.25	0.07
Isolation	1.91	0.45	1.71	0.40	2.78	0.68	0.89	0.21
Mindfulness	0.38	0.11	0.93	0.26	1.37	0.39	0.38	0.11
Over- Identification	2.35	0.56	1.49	0.35	2.78	0.68	0.57	0.13
**OMMP-8**	**Sex**		**Athlete Status**		**MH Diagnosis**		**Injury**	
Subfactor	MeanDiff	Cohen’s d	MeanDiff	Cohen’s d	MeanDiff	Cohen’s d	MeanDiff	Cohen’s d
Irreversible Pain	0.24	0.12	0.93	0.49	1.23	0.63	1.56	0.88
Emotional Flooding	1.06	0.34	1.11	0.37	2.00	0.68	0.79	0.26
Narcissistic Wounds	0.23	0.11	0.69	0.34	0.95	0.48	0.44	0.22

Note: SCS = Self-Compassion Scale; OMMP-8 = Orbach and Mikulincer Mental Pain Scale; MeanDiff = mean difference.

**Table 5:** SCS and OMMP-8 canonical correlation. Canonical correlation statistics for the SCS and OMMP-8.

	Correlation	Eigenvalue	Wilk’s λ	F	Sig.
CC Set 1	0.728	1.125	0.432	63.543	0.000
CC Set 2	0.245	0.064	0.919	10.111	0.000
CC Set 3	0.150	0.023	0.978	6.731	0.000

Note: CC = canonical correlation.

**Table 6:** Canonical loadings and coefficient values. SCS and OMMP-8 subfactor canonical correlation loadings and coefficient values.

Item Content	CC Set 1		CC Set 2		CC Set 3	
*SCS Subfactor*	Loading	Coefficient	Loading	Coefficient	Loading	Coefficient
Self-Kindness	0.608	−0.005	−0.478	−0.410	−0.161	−0.909
Self-Judgement	−0.913	−0.281	0.067	0.331	0.240	0.640
Common Humanity	0.403	0.023	−0.531	−0.399	−0.019	−0.404
Isolation	−0.911	−0.363	0.190	0.708	−0.151	−0.797
Mindfulness	0.554	0.067	−0.318	0.008	0.386	1.461
Over-identification	−0.936	−0.394	−0.303	−1.445	0.034	0.252
***OMMP-8 Subfactor***						
Irreversible Pain	−0.612	−0.090	0.420	0.467	−0.670	−1.117
Emotional Flooding	−0.939	−0.713	−0.337	−0.987	−0.065	0.033
Narcissistic Wounds	−0.769	−0.357	0.566	0.834	0.299	0.849

Note: CC = canonical correlation; SCS = Self-Compassion Scale; OMMP-8 = Orbach and Mikulincer Mental Pain Scale.

## Data Availability

Data can be made available upon reasonable request.
